# Burden of asymptomatic malaria among a tribal population in a forested village of central India: a hidden challenge for malaria control in India

**DOI:** 10.1016/j.puhe.2017.02.010

**Published:** 2017-06

**Authors:** M.K. Chourasia, K. Raghavendra, R.M. Bhatt, D.K. Swain, N. Valecha, I. Kleinschmidt

**Affiliations:** a National Institute of Malaria Research (ICMR), Kondagaon, Chhattisgarh, India; b National Institute of Malaria Research (ICMR), Dwarka, New Delhi, India; c National Institute of Malaria Research (ICMR), Lalpur, Raipur Chhattisgarh, India; d Department of Infectious Disease Epidemiology, London School of Hygiene and Tropical Medicine, London, UK

**Keywords:** Asymptomatic malaria, Parasitaemia, Chhattisgarh, India

## Abstract

**Objective:**

Chhattisgarh in India is a malaria-endemic state with seven southern districts that contributes approximately 50–60% of the reported malaria cases in the state every year. The problem is further complicated due to asymptomatic malaria cases which are largely responsible for persistent transmission. This study was undertaken in one of the forested villages of the Keshkal subdistrict in Kondagaon district to ascertain the proportion of the population harbouring subclinical malarial infections.

**Study design:**

Community-based cross-sectional study.

**Methods:**

Mass blood surveys were undertaken of the entire population of the village in the post-monsoon seasons of 2013 and 2014. Fingerprick blood smears were prepared from individuals of all ages to detect malaria infections in their blood. Individuals with fever at the time of the survey were tested with rapid diagnostic tests, and parasitaemia in thick blood smears was confirmed by microscopy. Malaria-positive cases were treated with anti-malarials in accordance with the national drug policy.

**Results:**

Peripheral blood smears of 134 and 159 individuals, including children, were screened for malaria infection in 2013 and 2014, respectively. Overall, the malaria slide positivity rates were 27.6% and 27.7% in 2013 and 2014, respectively, and the prevalence rates of asymptomatic malaria were 20% and 22.8%. This study showed that, for two consecutive years, the prevalence of asymptomatic malaria infection was significantly higher among children aged ≤14 years (34.4% and 34.1% for 2013 and 2014, respectively) compared with adults (15.2% and 18.2% for 2013 and 2014, respectively; *P* = 0.023 and 0.04, respectively).

**Conclusion:**

The number of asymptomatic malaria cases, especially *Plasmodium falciparum*, is significant, reinforcing the underlying challenge facing the malaria elimination programme in India.

## Highlights

• The study was carried out in a difficult-to-reach forested village with minimal access to healthcare facilities.
• There exists a large proportion of asymptomatic cases in the community.
• Children aged under 14 years comprised higher number of asymptomatic cases than adults.
• This underlines one of the potential challenges of public health in malaria control and elimination programs.

## Introduction

In India, the national programme reported 1.1 million new cases of malaria and 561 deaths in 2014. Two protozoan parasites—*Plasmodium vivax* and *Plasmodium falciparum*—lead to malaria deaths with a ratio of approximately 1:1.9. The state of Chhattisgarh, representing approximately 2% of the population of India, is a malaria-endemic state, accounting for nearly 12% of all reported cases. *P. falciparum* is the predominant (84%) parasite species, followed by *P. vivax*. There are also sporadic reports of *Plasmodium malariae* infections from southern districts (Dantewada, Bastar and Kondagaon), although these account for <1% of the cases in the district. There were 53 reported deaths due to malaria in Chhattisgarh state in 2014.^1^

The southern part of Chhattisgarh comprises seven administrative districts, namely Bijapur, Sukma, Dantewada, Bastar, Kondagaon, Narayanpur and Kanker (total population three million). These districts are mainly forested and inhabited by predominantly native tribal populations and have remained endemic for malaria since the inception of the national malaria control programme. In 2014, the annual parasite incidence (API, cases/1000 population/year) was 8.4 in one district (Kanker) and ranged from 10.4 to 53.6 in the other six districts. These districts, including Kondagaon, contributed 58.4% (total positive count 68,505/117,292) of all cases of malaria reported in Chhattisgarh state in 2014. Despite regular malaria control activities supported by a near-adequate health infrastructure, fortnightly active fever surveillance and treatment of malaria infection, indoor residual spraying with synthetic pyrethroids (coverage rate 44–88%) and distribution of long-lasting insecticidal nets (user rate 35–65%), there is persistent transmission of malaria in this area.^2^ The persistence of the infections could be due to asymptomatic cases. Asymptomatic (subclinical) malaria refers to the presence of malaria parasites in the blood without symptoms, usually provides a reservoir for transmission, and is an antecedent to symptomatic malaria.^3^ Previously, it was believed that only areas of high endemicity are at risk of subclinical infection, but more recent studies from other malaria-endemic regions (e.g. countries in Africa) have observed that communities living in low-transmission areas are also at the risk of asymptomatic parasitaemia.^4^ In low-transmission areas, submicroscopic carriers can become the source of approximately 20–50% of all transmission.^5^ The presence of a large number of asymptomatic carriers in a population is a challenge and places an additional burden on malaria control programmes.^6^

In India, studies in malaria-endemic areas have shown inconsistent findings in terms of the presence of asymptomatic malaria in the region, especially in tribal populations of Maharashtra, West Bengal and Assam.^3^, ^7^, ^8^, ^9^, ^10^ Seasonal transmission is another factor that influences the prevalence of subclinical parasitaemia in the community.^8^

Few published data on the burden of asymptomatic malaria parasitaemia are available from Chhattisgarh state. A cross-sectional survey carried out in 47 villages of Keshkal subdistrict of Kondagaon district in 2013 showed a high prevalence (slide positivity rate [SPR] 27.6%) of malaria in Randha village, and this was very high compared with the data provided by the state health department (10.3%), with a reported API of 3.4. A likely explanation for the disparity in reported cases is the presence of asymptomatic cases that may not have been captured through routine fever surveillance. Thus, reported incidence would underestimate the burden of malaria in the population. A correct estimate of symptomatic and asymptomatic cases is required to plan an effective malaria control programme. As such, this study was undertaken in 2014 to estimate the burden of asymptomatic malaria in a tribal population residing in Randha village.

## Methods

### Study area

This study was undertaken in Randha, a densely forested, hilly village (20° 05′18.32″N, 81°31′42.58″ E and elevation 653 m) in Kondagaon district, which is one of the malaria-endemic districts in the Chhattisgarh state. Randha village has 220 inhabitants, belonging predominantly to the aboriginal ‘Gond’ tribe. The village is in a forested area, is not connected by roads that are accessible in all weathers, and is 12 km from the nearest health facility (Keshkal Community Health Centre [CHC]). Precipitation (average 1338 mm) occurs mainly between June and September. Subsistence agriculture and collection and selling of forest produce are the main sources of livelihood. *Anopheles culicifacies* is the main malaria vector and is reported to be resistant to DDT, malathion and synthetic pyrethroid alpha-cypermethrin, which was introduced into the malaria control programme a decade ago.^11^ Fortnightly house-to-house visits for fever surveillance are conducted by paramedical staff of a health subcentre located in a nearby village. Rapid diagnostic tests (RDT; SD BIOLINE Malaria Ag *Pf/Pv*, Standard Diagnostics Inc., Gurgaon, India; sensitivity *P. falciparum* ≥99.7%, *P. vivax* 95.5%; specificity 99.5%) are performed, and blood slides collected from patients with fever are sent to the CHC for microscopic examination. Malaria-positive cases are treated with anti-malarial drugs in accordance with the national guidelines. Health staff cannot reach the village regularly to provide health care, and villagers cannot seek health care in time for prompt treatment at the CHC. Due to poor accessibility and the shortfall of resources, anti-malarial activities are not satisfactory. The API for the last five years varies between one and five cases/1000 population/year using the data available at the CHC.

### Data collection

Blood surveys were undertaken during the post-monsoon seasons of 2013 and 2014. The two surveys were undertaken during the same season for two consecutive years to avoid the confounding factor of seasonal variation in the study. All the inhabitants of Randha village were included in the study. A census was undertaken by the survey teams, and all the houses in the village were enumerated. The objectives and purpose of the study were explained to all the households. Written informed consent was acquired from the head of each household willing to participate in the study after reading and explaining the content of the information sheet. Blood slides were collected from all inhabitants available in the household at the time of the survey, regardless of the presence of fever. Axillary temperature was recorded using a digital thermometer. In addition to the collection of blood slides, individuals with fever were tested for malaria using a bivalent RDT. For each study participant, a finger prick was performed and thick and thin blood smears were prepared. Slides were taken to a field laboratory, stained with Giemsa and examined for the presence of malaria parasites by trained technicians. Parasite density was calculated against 200 white blood cells. *P. falciparum* cases (RDT and slide positives) were treated with artesunate + sulfadoxine-pyrimethamine (artesunate combination therapy), and *P. vivax* cases were treated with chloroquine and primaquine by trained project staff in accordance with the national drug policy.

As a standard protocol, all positive slides and 10% of randomly selected negative blood slides were cross-examined by independent laboratory technicians to monitor examination quality. RDT results were blinded to the microscopist who had examined the corresponding blood slides of the participants.^12^

A quality check of RDT kits was undertaken before their distribution to field workers, and an internal quality check for reading RDT results was made by two independent workers and subsequently confirmed. In cases of any discordance, the trained field supervisor re-examined the results independently. All RDT kit results were cross-examined with the slide results routinely for any significant deviation in the results, especially for false-positive cases.

Although this study planned to include all village inhabitants (at the time of the survey) in the 2013 survey, a few children and women refused to give their consent to participate due to apprehension about blood slide preparation. In the 2014 survey, the participation rate increased following continuous healthcare orientation and counselling by project staff.

### Study definition and statistical analysis

A case of fever was defined as an individual with a history of fever within the past 48 h or a current temperature ≥37.5 °C. An asymptomatic case of malaria was defined as absence of fever within the past two weeks, the previous night and at the time of survey, and the presence of malaria parasites in peripheral blood as determined by microscopic examination of the blood smear. All the study data, clinical data and laboratory results were entered and analysed in Excel 7 (Microsoft Corp, Redmond, WA, USA) and SPSS version 16 (IBM Corp., Armonk, NY, USA), respectively. All continuous variables such as age were expressed as a mean and standard deviation. Cross-tabulation was performed for categorical variables. Chi-squared (χ^2^) test was applied to assess the association between two variables.

## Results

Two consecutive surveys were undertaken in the post-monsoon seasons of 2013 and 2014, and 134 and 159 individuals of all age groups (Table 1) were included, respectively. The mean age of study participants was 28 years (median 30 years). Children accounted for 27.6% and 30.2% of the study population in 2013 and 2014, respectively.

**Table 1:** Characteristics of study population (2013, *n* = 134; 2014, *n* = 159).

Variable	Asymptomatic		Symptomatic	
	*P. falciparum*	*P. vivax*	*P. falciparum*	*P. vivax*
**2013**				
Percentage (*n* = 134)	12.6 (17)	5.2 (7)	8.2 (11)	1.5 (2)
Age, mean (years; range)	23.3 (4–65)	18 (4–34)	15.5 (1–79)	30 (15–45)
Males, *n* (%)	9 (52.9)	7 (100)	7 (63.6)	2 (100)
Parasite density, geometric mean (μl, range)	412 (80–27,040)	287 (120–2120)	2354 (80–205,600)	1187 (800–1760)
**2014**				
Percentage (*n* = 159)	20.9 (31)	1.3 (02)	6.9 (11)	Nil
Age, mean (years; range)	24 (1–71)	25.5 (20–31)	16 (1–45)	ND
Males, *n* (%)	14 (45.2)	0	4 (36.4)	ND
Parasite density, geometric mean (μl, range)	849 (40–207,840)	23 (16–32)	740 (120–6120)	ND

Abbreviations: *P. falciparum*, *Plasmodium falciparum*; *P. vivax*, *Plasmodium vivax*; ND, not done.

With slide positivity rates of 27.6% (37 positives out of 134 blood smears examined) in 2013 and 27.7% (44/159) in 2014, the proportion of *P. falciparum* infections was higher in both years (75.7% in 2013 and 95.5% in 2014; Table 1).

The geometric mean (range) of the parasite density of asexual stages in symptomatic and asymptomatic *P. falciparum* cases were 2354 (80–205,600) and 849 (40–207,840), respectively. Similarly, the parasite density of asexual stages in symptomatic and asymptomatic *P. vivax* cases were 1187 (800–1760) and 287 (120–2120), respectively (Table 1) [2013, *t*-test 2.34 (*P* = 0.009); 2014, *t*-test 0.14 (*P* = 0.89)].

**Fig. 1:**
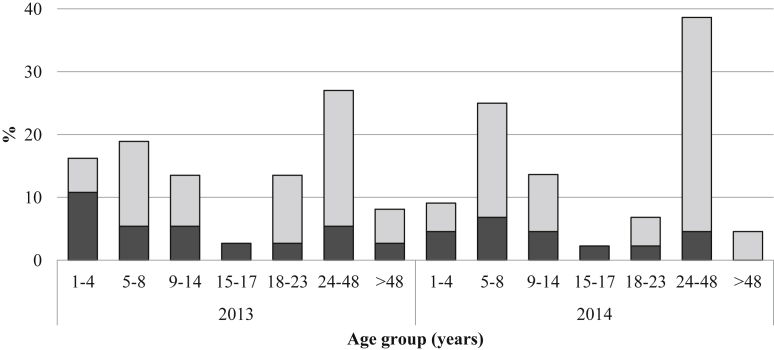
Malaria-positive cases among respondents of different age groups presenting with and without fever in 2013 and 2014. Black bars, symptomatic; grey bars, asymptomatic.

The proportion of symptomatic patients was 9.7% (13/134) in 2013 and 6.9% (11/159) in 2014, and the proportion of asymptomatic patients was 17.9% (24/134) and 20.7% (33/159) in 2013 and 2014, respectively (Table 2). The study indicated a higher proportion of *P. falciparum* infection among both symptomatic and asymptomatic cases. Comparison of asymptomatic parasitaemia between different age groups indicated a higher proportion among adults aged 24–48 years in both 2013 and 2014 (Fig. 1).

**Table 2:** Proportion of asymptomatic malaria cases among children and adults (2013, *n* = 134; 2014, *n* = 159).

Year	Variable	All subjects		Odds ratio (95% CI)	Asymptomatic subjects		Odds ratio (95% CI)
		Blood slides examined	Positives (%)		Blood slides examined	Positives (%)	
2013	Children (age ≤14 years)	37	18 (13.4)	3.9 (1.7–8.8), *P* = 0.01	29	10 (8.2)	2.9 (1.1–7.6), *P* = 0.023
	Adults	97	19 (14.2)		92	14 (11.6)	
	Total	134	37 (27.6)		121	24 (19.8)	
2014	Children (age ≤14 years)	48	21 (13.2)	2.9 (1.4–6.2), *P* = 0.003	41	14 (9.7)	2.3 (1.03–5.24), *P* = 0.04
	Adults	111	23 (14.5)		104	19 (13.1)	
	Total	159	44 (27.7)		145	33 (22.8)	

Abbreviation: CI, confidence interval.

Surveys in 2013 and 2014 showed that the odds of presentation of asymptomatic malaria in children (age ≤14 years) were, respectively, 2.9 times (95% confidence interval [CI] 1.1–7.6) and 2.3 times (95% CI 1.0–5.2) higher compared with adults (age >14 years; *P* < 0.01). The odds of presentation of symptomatic malaria in these age groups were 3.9 times (2013: 95% CI 1.7–8.8) and 2.9 times (2014: 95% CI 1.4–6.3, *P* < 0.01) higher compared with adults (Table 2).

Regression of parasite density and age of patients showed inverse but weak correlations [Y = 21.64–9.78×, *R*^2^ = 0.058 (*P* = 0.151); Y = 22.5–6.3×, *R*^2^ = 0.058 (*P* = 0.303)] between parasite density and age. With an increase in age, there was a decrease in parasite density among the malaria-positive subjects.

## Discussion

Surveillance data from Keshkal CHC showed seven malaria-positive cases out of 68 slides (SPR 10.3%) in 2013, and eight malaria-positive cases out of 80 slides (SPR 10%) in 2014. Mass blood surveys undertaken in the post-monsoon seasons of 2013 and 2014 in the same village revealed that a large number of individuals were harbouring malaria parasites asymptomatically and were not captured in the routine fever surveillance. Also, the incidence of malaria reported routinely by the healthcare system revealed a lower number of symptomatic malaria cases compared with these mass surveys. A huge gap has been observed previously between the incidence of malaria reported during routine surveillance by the primary healthcare system and that reported by parallel longitudinal studies carried out in some states of India: 68%–98% between the reported and true incidence of malaria.^13^ Similarly, this study observed that the actual morbidity due to malaria in the community was up to three times higher than the reported incidence. In the present study, the asexual parasite density of both *P. falciparum* and *P. vivax* was considerably higher in symptomatic cases compared with asymptomatic cases in 2013. A study in Kenya also reported less asexual parasite density among the asymptomatic carriers.^14^ However, in surveys performed in the same population in 2014, no significant difference was observed between the asexual *P. falciparum* parasite density in symptomatic and asymptomatic malaria cases. This major difference in the upper limit of the range was due to one child who presented with asymptomatic malaria and a parasite density of 207,840 parasites/μl. If this child is considered as an outlier, the range would become 40–8600 p/μl in 2014, which is comparable with the parasite density of symptomatic patients in 2014.

There are different views on the clinical consequences of asymptomatic malaria and its effect on immunity. First, long-term asymptomatic carriage can be protective against malaria; this may be due to the acquired immunity of the individual^15^, ^16^, ^17^ and could be self-limiting.^18^ Asymptomatic cases could be due to partial immunity, which is mainly due to inhibition of parasite toxin(s)-mediated cytokine responses.^19^, ^20^, ^21^ The present study revealed that the odds of being asymptomatic were higher among children than adults, suggesting that age could be an important risk factor for the presence of subclinical infections. Similarly, other studies have reported that children can act as a parasitic reservoir and facilitate the transmission of malaria. This may have serious implications in young children, as it can affect brain development and academic performance,^22^, ^23^ and parasitaemia can lead to severe malaria.^24^, ^25^ Other plausible harmful effects include a decrease in iron absorption and impairment of cognitive function.^26^

Various strategies have been suggested such as intermittent preventive treatment and periodic mass parasite screening to eliminate the foci of asymptomatic carriers in the population.^25^ Thus, regular surveillance and mass blood screening of children aged ≤14 years are important.^27^

There is a need for more follow-up cohort studies with representative samples to identify the role of asymptomatic cases in the dynamics of malaria transmission, seasonal variability and its contribution to the incidence of malaria in the region, and support in planning an effective and appropriate malaria elimination strategy.

In conclusion, this study found a large proportion of asymptomatic cases in the community, including children, which can be attributed to immunity. Asymptomatic malaria cases are not captured in routine fever surveillance and can develop into clinical malaria with severe symptoms. Thus, despite its small sample size, this study clearly highlights the underlying challenge facing the national malaria control programme in its efforts towards malaria elimination.

## Author statements

### Acknowledgements

The authors wish to thank all the participants for their time and involvement in the study, all the field staff and laboratory technicians for their support in data collection and slide examination, and NIMR, New Delhi for constant support.

### Ethical approval

Ethical approval for this study was obtained as a part of the IIR-India project from the Institutional Ethical Committee of National Institute of Malaria Research, New Delhi, India (ECR/NIMR/EC/2010/75).

### Funding

Funding was provided by the Bill and Melinda Gates Foundation (grant no. OPP 1062754). This research forms part of a multicountry study co-ordinated by the Global Malaria Programme of the World Health Organization. The funding agency had no role in the planning, study design, data collection or write up.

### Competing interests

None declared.
